# Professional Roles and Work-Related Challenges of Anti-Drug Social Workers in Community-Based Drug Rehabilitation: A Systematic Review

**DOI:** 10.3390/healthcare14131849

**Published:** 2026-06-25

**Authors:** Wang Jianping, Paramjit Singh Jamir Singh, Azlinda Azman

**Affiliations:** Social Work Section, School of Social Sciences, Universiti Sains Malaysia, Minden 11800, Penang, Malaysia; 2720349866jianping@student.usm.my

**Keywords:** anti-drug social workers, community-based drug rehabilitation, public health workforce, professional roles, occupational stress, work challenges, social work practice

## Abstract

**Background/Objectives:** Community-based drug rehabilitation is a key component of public health strategies in China, with anti-drug social workers playing a frontline role in relapse prevention, social reintegration, and long-term recovery. However, the sustainability and effectiveness of this workforce remain uncertain due to complex organisational and structural conditions. This study aims to examine the professional roles, work-related challenges, and coping strategies of anti-drug social workers within community-based rehabilitation systems. **Methods:** A systematic review was conducted in accordance with PRISMA 2020 guidelines and was registered in PROSPERO (Registration ID: 1381833). The literature published between 2009 and 2025 was identified through Google Scholar, PubMed, Web of Science, and the Electronic Library. A total of 35 Chinese and English-language studies met the inclusion criteria and were analysed to synthesise evidence on social work practice in drug rehabilitation contexts. **Results:** The findings identify three core professional roles: information provider, resource linker, and relationship repairer. These roles highlight the multifaceted contribution of social workers in bridging institutional systems and client needs. However, their effectiveness is constrained by fragmented governance structures, role conflict, professional identity ambiguity, administrative burden, limited training, and sustained emotional labour. These conditions contribute to occupational stress, burnout risk, and workforce instability, which weaken service continuity and client-centred care. **Conclusions:** Strengthening community-based drug rehabilitation requires addressing workforce and system-level constraints. Clearer role definition, targeted interdisciplinary training, reduced administrative demands, and structured organisational support are essential to enhance professional capacity, improve service delivery, and support long-term recovery outcomes.

## 1. Introduction

Drug abuse has wide-ranging consequences for individuals, families, and communities, including physiological, psychological, economic, and social harm. In China, the number of people who use drugs remains high, and drug use is often concealed and difficult to address. Drug addiction is widely recognised as a chronic, relapsing, and complex brain disease [1]. It is associated with compulsive drug-seeking behaviour and persistent use despite harmful consequences, leading to significant physical, cognitive, emotional, and behavioural changes [2].

To sustain drug use, some individuals engage in illegal activities such as theft, robbery, or deception. Drug use may also be accompanied by hallucinations, impaired judgement, and aggressive behaviour, which can place both users and others at risk. During withdrawal, some individuals engage in self-harming behaviour in an attempt to relieve physical distress, resulting in lasting injury and chronic health problems. Repeated cycles of drug use, rehabilitation, and relapse gradually erode family trust and contribute to strained relationships, social isolation, and family breakdown [3].

In China, conventional approaches to drug rehabilitation have largely prioritised physical detoxification, with limited emphasis on psychological recovery, social reintegration, and long-term support. This imbalance increases the likelihood of relapse and produces broader social and public health consequences [4,5]. Evidence suggests that reliance on compulsory detoxification alone has limited effectiveness, particularly in supporting sustained recovery. Beyond medical treatment, individuals undergoing rehabilitation face significant challenges in rebuilding daily functioning and reintegrating into community life.

Anti-drug social workers play a key role in addressing these challenges within community-based drug rehabilitation systems. Guided by professional values, ethical principles, and specialised knowledge, social workers provide a range of services including daily life support, rehabilitation assistance, employment guidance, legal consultation, and behavioural supervision [6]. However, the delivery of these services often exposes anti-drug social workers to substantial occupational pressure, emotional strain, and personal risk [7,8,9]. These challenges are critical to address because the effectiveness of rehabilitation services is closely linked to the wellbeing, stability, and capacity of the workforce.

From a professional perspective, anti-drug social workers view people who use drugs as individuals in need of support rather than as offenders or patients. In practice, however, social workers are frequently required to assume additional roles such as administrative assistants, drug control coordinators, and stability maintenance personnel [10]. This expansion of responsibilities contributes to role ambiguity, ethical tension, and difficulties in maintaining professional boundaries. A comprehensive understanding of the professional roles, work challenges, and coping strategies of anti-drug social workers is therefore necessary to strengthen workforce support and improve the quality of community-based drug rehabilitation services. This systematic review examines the roles challenges and coping strategies of anti-drug social workers within community-based drug rehabilitation contexts.

## 2. Materials and Methods

A systematic literature search was conducted to identify studies published between 2009 and 2025 using Google Scholar, PubMed, Web of Science, and the Electronic Library. Google Scholar was included to maximise the identification of relevant studies and grey literature that may not have been indexed in traditional academic databases. To maintain feasibility and relevance, records were screened according to their order of relevance as generated by the search engine. Titles and abstracts were reviewed against the predefined eligibility criteria, and only studies directly related to anti-drug social work practice within community-based rehabilitation settings were retained for further assessment. The review followed the PRISMA 2020 guidelines and applied a structured search and screening process to ensure transparency, rigour, and relevance in study selection [8,11,12,13,14,15,16,17,18,19]. It was also registered in PROSPERO (Registration ID: 1381833). In addition, references cited in relevant review articles were manually screened to reduce the risk of missing eligible studies.

Two reviewers independently screened all titles and abstracts according to the predefined inclusion and exclusion criteria. Full-text articles were subsequently assessed for eligibility. Any disagreements between reviewers were discussed and resolved through consensus. Where consensus could not be reached, consultation with a third reviewer was undertaken. This process ensured consistency and reduced potential selection bias during study identification and eligibility assessment.

The search strategy was developed to capture literature relating to anti-drug social work practice within community-based drug rehabilitation settings. Search terms were organised into three conceptual categories: (1) anti-drug social work and rehabilitation services, (2) professional roles and work-related experiences, and (3) coping strategies and workforce development. Keywords included “anti-drug social worker”, “drug rehabilitation social work”, “community-based drug rehabilitation”, “community detoxification”, “substance use rehabilitation”, “professional role”, “work challenge”, “occupational stress”, “workforce development”, and “coping strategy”. Boolean operators (AND, OR) were used to combine search terms across databases to improve search sensitivity and specificity.

Studies were included if they met the following criteria:

(a) publications written in English;
(b) publications written in Chinese;
(c) studies addressing the professional roles of anti-drug social workers;
(d) analyses of anti-drug social work practice;
(e) studies examining the working conditions or professional experiences of anti-drug social workers;
(f) research related to the development or construction of the anti-drug social worker workforce.

Studies were excluded if they:

(a) provided only theoretical discussions of community detoxification models;
(b) focused primarily on community-level drug prevention and control systems rather than social work practice;
(c) involved non-randomised control experiments;
(d) examined general helping or educational services without a specific focus on anti-drug social workers.
(e) non-peer-reviewed materials lacking sufficient methodological information, including editorials, opinion pieces, commentary articles, newsletters, and informal online publications.

Grey literature, including master’s dissertations and conference papers, was considered for inclusion when it addressed the professional roles, work-related challenges, or coping strategies of anti-drug social workers and provided sufficient methodological detail. The inclusion of grey literature was intended to capture emerging evidence and reduce publication bias. Editorials, commentaries, opinion papers, newspaper articles, and non-academic online materials were excluded from the review.

All retrieved records were imported into EndNote 20 for reference management. Duplicate records identified across databases were automatically detected and subsequently verified through manual checking. Duplicates were removed prior to title and abstract screening to ensure that each study was assessed only once during the review process.

A thematic synthesis approach was employed. Extracted findings were coded, compared, and grouped into recurring themes. Through iterative analysis, themes were organised into three professional roles, work-related difficulties, and coping strategies. Data extraction was conducted independently by two reviewers using a structured data extraction form. Information extracted from each study included author details, publication year, study design, study setting, participant characteristics, professional roles of anti-drug social workers, work-related challenges, coping strategies, and key findings. Extracted data were reviewed and compared to ensure accuracy and completeness. The findings were then synthesised using a thematic analysis approach, whereby similar concepts and recurring patterns were grouped into broader themes relating to professional roles, work-related challenges, and coping strategies.

The search process aimed to synthesise evidence on the professional roles, work challenges, and coping strategies of anti-drug social workers in practice. The initial search yielded 308 records, including peer-reviewed journal articles, master’s theses, conference papers, and related publications. All records were imported into EndNote 20 for data management and screening. After the removal of duplicates and irrelevant records, 35 publications were retained for final analysis. Among these, 3 studies focused on literature retrieval, 14 examined work-related difficulties faced by anti-drug social workers, 11 addressed anti-drug social work practice, 4 focused on workforce development, and 3 examined broader drug-related research topics.

The methodological quality of the included studies was assessed using the Joanna Briggs Institute (JBI) Critical Appraisal Checklist appropriate to each study design. Two reviewers independently evaluated the methodological rigour of all included studies. Any differences in assessment were discussed and resolved through consensus. The appraisal focused on the clarity of research objectives, appropriateness of the research design, adequacy of data collection procedures, rigour of data analysis, credibility of findings, and overall methodological transparency. The quality assessment was conducted to enhance the trustworthiness of the review findings and to inform the interpretation of the evidence.

**Figure 1:**
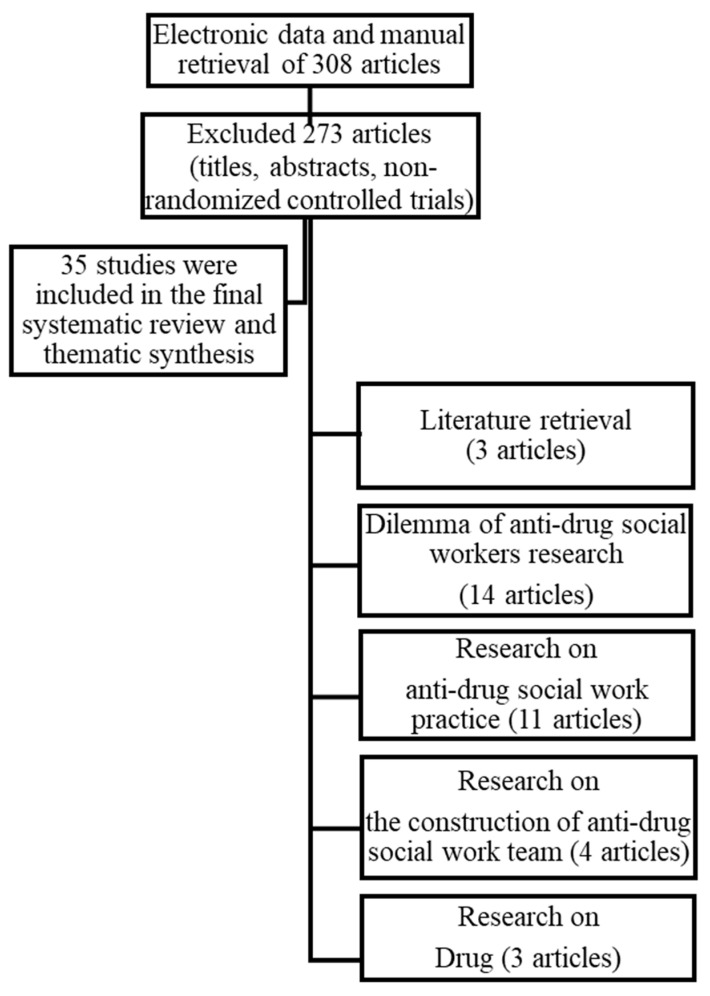
Flow Chart of the Systematic Literature Review.

Following duplicate removal and eligibility screening, 35 studies met the inclusion criteria and were included in the final thematic synthesis. Studies excluded during screening consisted primarily of duplicate records, publications unrelated to anti-drug social work practice, theoretical discussions without empirical evidence, and non-peer-reviewed materials that did not meet the review criteria (Figure 1).

## 3. Results

This systematic review employed thematic synthesis to integrate evidence on the professional roles, work-related challenges, and coping strategies of anti-drug social workers across the included studies. According to the reviewed studies, the findings highlight not only the breadth of anti-drug social work practice but also the structural and occupational conditions that shape how services are delivered in real-world settings. The evidence reflects a profession that operates within complex systems, where social workers are required to respond to diverse client needs while navigating organisational expectations and resource constraints. Their work is both multifaceted and demanding, illustrating the critical yet often under-recognised contribution of social workers within drug control and rehabilitation frameworks.

For clarity, the results are organised into two main components: the core professional roles performed by anti-drug social workers and the work-related difficulties encountered in practice. This structure allows for a more focused understanding of both what social workers do and the challenges they face in carrying out these responsibilities. By examining these two dimensions together, the review provides a more comprehensive picture of anti-drug social work, highlighting the interaction between role expectations and workplace realities.

### 3.1. Quality Assessment of Included Studies

The quality appraisal indicated that most of the included studies demonstrated satisfactory methodological quality. The majority clearly described their research objectives, study design, participant characteristics, and data collection procedures. Several studies also provided detailed explanations of their analytical approaches and findings. However, some studies offered limited information regarding researcher reflexivity, procedures to minimise potential bias, and strategies used to ensure the credibility and trustworthiness of the findings. Despite these limitations, all included studies were considered sufficiently relevant and methodologically sound to contribute to the thematic synthesis. Overall, the evidence base provides a reasonable level of confidence in the findings related to the professional roles, work-related challenges, and coping strategies of anti-drug social workers.

### 3.2. Roles of Anti-Drug Social Workers

National policy frameworks consistently recognise anti-drug social workers as a key professional group within drug control and rehabilitation systems. Policy initiatives, such as the proposal to establish a workforce of 100,000 anti-drug social workers and the issuance of the Opinions on Strengthening the Construction of the Team of Anti-Drug Social Workers in 2017, reflect increasing institutional attention to the role of professional social work in this field [7]. Policy initiatives, such as the proposal to establish a workforce of 100,000 anti-drug social workers and the issuance of the Opinions on Strengthening the Construction of the Team of Anti-Drug Social Workers in 2017, reflect increasing institutional attention to the role of professional social work in this field.

Despite these policy advances, important challenges remain. Although national statistics indicate a sustained decline in registered and newly identified drug users, the reviewed literature highlights a persistent gap between short-term control outcomes and long-term recovery effectiveness. In particular, high relapse rates following compulsory isolation rehabilitation suggest that existing approaches may not adequately support sustained reintegration into society [20,21]. The reviewed literature highlights a persistent gap between short-term control outcomes and long-term recovery effectiveness, particularly high relapse rates following compulsory isolation rehabilitation.

Within this policy environment, anti-drug social workers operate at the intersection of regulatory control and rehabilitative support. Their work is shaped by institutional mandates that emphasise monitoring and compliance, while also requiring them to deliver client-centred services grounded in professional ethics. The reviewed studies indicate that social workers must balance these sometimes-competing demands while upholding core values such as respect, objectivity, fairness, and impartiality. The reviewed studies indicate that social workers must balance regulatory responsibilities and client-centred services while upholding professional values.

Across different stages of rehabilitation, social workers assume multiple roles in response to diverse client needs and organisational expectations. The literature consistently identifies three core professional roles: information provider, resource linker, and relationship repairer [6]. As information providers, social workers offer guidance on treatment processes, legal obligations, and health-related issues. In their role as resource linkers, they connect individuals to relevant services, including healthcare, employment support, and community resources. As relationship repairers, they help rebuild trust between clients, their families, and wider social networks, which is often critical for long-term recovery.

In practice, these roles are rarely performed in isolation. Instead, they frequently overlap, requiring social workers to move fluidly between different functions within a single case or interaction. This overlap reflects both the complexity of clients’ situations and the constraints of organisational settings, where limited resources and high caseloads are common. As a result, anti-drug social workers must demonstrate flexibility, resilience, and strong professional judgement to effectively manage their responsibilities and support meaningful rehabilitation outcomes.

### 3.3. Information Provider

As information providers, anti-drug social workers play a central role in reducing information asymmetry between institutions and clients. The reviewed studies show that social workers routinely translate complex systems into accessible knowledge, helping clients better understand the frameworks that govern their rehabilitation. This includes providing guidance on laws, regulations, employment policies, and programme requirements, ensuring that individuals are aware of both their rights and obligations within the rehabilitation process [22,23]. Social workers provide information on laws, regulations, employment policies, programme requirements, and rehabilitation procedures.

A key aspect of this role involves explaining technical and often confusing policy provisions. These may include dynamic management procedures, hair testing requirements, and reporting obligations, all of which can be difficult for clients to interpret without support. The literature highlights that misunderstanding these requirements can lead to non-compliance, which may have serious consequences for individuals in rehabilitation. By offering clear, consistent, and personalised explanations, social workers help reduce confusion and enable clients to engage more effectively with institutional processes.

Many individuals’ undergoing rehabilitation face significant physical and mental health challenges, alongside elevated stress levels and reduced social capital resulting from prolonged drug use [24,25]. These factors often limit their access to stable employment and broader social resources. Within this context, anti-drug social workers extend their informational role to include practical guidance on employment pathways. They provide up-to-date labour market information, clarify recruitment criteria, and help clients identify job opportunities that align with their current abilities and circumstances.

In addition to sharing information, social workers often support clients in interpreting and applying this knowledge in meaningful ways. For individuals with disrupted work histories or limited formal education, understanding how to search for jobs, prepare applications, or meet employer expectations can be particularly challenging. The reviewed studies suggest that social workers play an important role in breaking down these processes into manageable steps, thereby increasing clients’ confidence and readiness to engage with the labour market.

Through sustained informational support, social workers facilitate gradual workforce reintegration while also strengthening clients’ understanding of broader social norms and expectations. This function is especially important for individuals with low educational attainment or limited experience navigating formal employment systems [26,27]. By equipping clients with relevant knowledge and practical skills, social workers help them rebuild a sense of agency and direction during recovery.

Overall, the role of information provider extends beyond simply delivering facts. It involves ongoing engagement, clarification, and reinforcement, tailored to the evolving needs of each individual. By improving access to accurate and relevant information, anti-drug social workers help reduce uncertainty, enhance decision-making capacity, and support greater confidence throughout the rehabilitation process.

### 3.4. Resource Linker

The role of resource linker emerges as a core function across the reviewed studies, highlighting the practical and coordination-focused dimension of anti-drug social work. Anti-drug social workers play a key role in connecting rehabilitation clients to welfare assistance, rehabilitation services, healthcare resources, and community support systems [28,29]. This function is particularly critical given the multiple and overlapping disadvantages faced by many clients, including unstable housing, poor health, and financial insecurity. By identifying available resources and facilitating access, social workers help stabilise clients’ living conditions, creating a more supportive foundation for recovery.

A central component of this role involves assisting eligible clients in applying for minimum living security and other forms of social assistance. The reviewed literature shows that social workers actively guide clients through what can often be complex and bureaucratic processes. This includes helping them understand eligibility criteria, prepare necessary documentation, and communicate effectively with welfare authorities. Through this support, immediate material needs such as housing, food, and basic living expenses can be addressed, thereby reducing survival-related stress that might otherwise undermine rehabilitation efforts.

Beyond material assistance, anti-drug social workers also act as intermediaries between clients and specialised service providers. They facilitate access to legal services, psychological counselling, and family support to help address legal disputes, emotional distress, and strained interpersonal relationships [30,31]. This intermediary role is especially important for clients who may lack confidence, literacy, or familiarity with formal service systems. For individuals with repeated relapse histories, the literature further emphasises the importance of referral to voluntary detoxification programmes that integrate physical, psychological, and spiritual support. Social workers not only identify suitable programmes but also explain procedures, encourage participation, and interpret policies related to compulsory isolation and detoxification to support compliance and reduce relapse risk.

Health-related resource linkage represents another key dimension of this role. Many individuals in rehabilitation experience chronic illness, infectious diseases, or mental health conditions, often without adequate access to medical care. The reviewed studies indicate that anti-drug social workers assist clients in identifying appropriate healthcare providers, facilitating access to treatment, and ensuring continuity of care over time. By bridging gaps between clients and healthcare systems, social workers contribute to improved health management and more stable rehabilitation outcomes, reinforcing the broader goal of long-term recovery [32,33,34].

### 3.5. Relationship Repairer

Relationship repair constitutes a significant dimension of anti-drug social work practice, reflecting the deeply social nature of both addiction and recovery. The reviewed literature indicates that drug use frequently disrupts parent–child, spousal, and broader social relationships, often leaving individuals isolated and disconnected from key sources of support. Among these, parent–child relationships are most commonly affected, typically due to long-standing dysfunctional family dynamics, unresolved conflict, and repeated relapse experiences [35,36]. Over time, prolonged drug use erodes trust, weakens parental authority, and alters established family roles, resulting in patterns of avoidance, hostility, or emotional disengagement within households.

To address these complex relational challenges, anti-drug social workers employ family-centred intervention approaches that focus on restoring communication and rebuilding trust. The reviewed studies report the use of structural family therapy techniques to clarify blurred or overly rigid boundaries, reorganise family subsystems, and modify maladaptive interaction patterns [37,38,39]. Through guided dialogue and structured interventions, social workers help family members better understand each other’s perspectives and renegotiate roles in more constructive ways. These efforts are aimed at fostering a more stable and supportive family environment, which is widely recognised as a key factor in sustaining rehabilitation outcomes.

Marital relationships are also frequently damaged by prolonged drug use, with many individuals experiencing rejection, loss of trust, and emotional distancing from their partners. In some cases, these strains lead to separation or divorce, further reducing the individual’s support network [40,41]. The reviewed literature shows that social workers often intervene as case managers in these situations, facilitating communication between partners, clarifying expectations, and supporting gradual trust-building. This process is often slow and requires sensitivity, as both partners may carry emotional wounds and uncertainty about the future.

Beyond the immediate family context, anti-drug social workers also play an important role in helping clients repair relationships in the workplace and the wider community. Drug use is commonly associated with social exclusion, stigma, and the breakdown of social networks, which can make reintegration particularly challenging [42,43,44,45,46,47,48]. Social workers support clients in addressing interpersonal conflicts, rebuilding professional relationships, and developing the social skills needed to re-engage with others. These efforts often involve both direct intervention and ongoing encouragement, helping clients regain confidence in social interactions.

Social workers assist clients in rebuilding families, workplace, and community relationships. The reviewed studies suggest that relationship repair is not a one-time intervention but an ongoing process that evolves alongside the client’s rehabilitation journey. By working across family, workplace, and community contexts, anti-drug social workers contribute to rebuilding the social fabric surrounding individuals, ultimately supporting more sustainable reintegration and improved quality of life.

### 3.6. Emerging Work Difficulties

Despite their central role, the reviewed studies consistently document substantial work-related difficulties faced by anti-drug social workers [49,50,51]. One of the most frequently reported challenges is the complexity of being managed by multiple authorities, including social work agencies, anti-drug offices, community organisations, and public security departments. These overlapping governance structures often generate competing task demands, unclear reporting lines, and inconsistent performance expectations. As a result, social workers commonly experience role ambiguity and reduced professional autonomy, which can constrain their ability to prioritise client-centred practice and make independent professional judgements.

Expanding administrative workloads further intensify these challenges and place additional pressure on already stretched practitioners [52,53,54]. Social workers are often required to complete extensive documentation, data reporting, and ad hoc administrative assignments mandated by supervisory bodies. While these tasks are necessary for accountability and monitoring, they consume a significant proportion of working time. Consequently, opportunities for direct engagement with clients are reduced, and core professional activities such as assessment, counselling, and follow-up interventions may be compressed or delayed, ultimately affecting the depth and continuity of services.

Limited access to specialised training also emerges as a major constraint within the reviewed literature. Many anti-drug social workers report insufficient preparation in key areas such as psychology, psychiatry, infectious disease management, and legal procedures [55,56]. Given the complex and often co-occurring health and psychosocial issues faced by rehabilitation clients, these knowledge gaps can undermine practitioners’ confidence and effectiveness. This is particularly evident when responding to mental health crises, managing medical needs, or navigating legal risks. The absence of structured and ongoing professional development opportunities further exacerbates feelings of inadequacy and limits skill enhancement over time.

Clients often present with complex and overlapping needs that require support across several areas, including employment, healthcare, family relationships, and social reintegration. In practice, social workers are expected to respond to these needs within constrained environments where time and resources are limited. High caseloads reduce the amount of attention that can be given to each individual, while organisational pressures shape how interventions are prioritised and delivered. These conditions make it difficult to sustain continuous engagement with clients and may affect the depth of support provided. Structural limitations, including restricted access to employment opportunities and healthcare services, further influence the effectiveness of interventions and shape the outcomes that can realistically be achieved [57].

Emotional labour and psychological strain represent another prominent category of work difficulty. Anti-drug social workers are regularly exposed to challenging client behaviours, including relapse, resistance, aggression, and emotional instability. At the same time, they are expected to maintain professional boundaries and emotional neutrality, which can be difficult to sustain over long periods. Repeated exposure to these conditions places workers at increased risk of stress, emotional exhaustion, and burnout. The literature consistently highlights challenges related to emotional regulation, psychological adaptation, and maintaining long-term engagement with clients as critical occupational concerns [58,59,60,61].

Taken together, these findings illustrate that the difficulties faced by anti-drug social workers are not isolated issues but interconnected challenges embedded within broader organisational and structural contexts. The combination of administrative burden, limited training, complex client needs, and sustained emotional demands creates a high-pressure work environment. Addressing these issues requires not only individual coping strategies but also systemic improvements, including clearer role definitions, enhanced professional support, and more balanced workloads to enable effective and sustainable practice.

### 3.7. Coping Strategies Adopted by Anti-Drug Social Workers

The reviewed studies indicate that anti-drug social workers employ a range of coping strategies to manage occupational stress, emotional strain, role ambiguity, and organisational pressures. These coping mechanisms operate at emotional, professional, organisational, and personal levels, reflecting the complex nature of anti-drug social work practice.

One of the most frequently reported coping strategies is the utilisation of peer support networks. Social workers often rely on colleagues to share experiences, discuss difficult cases, and seek practical advice when facing challenging situations [7]. Informal peer consultation provides opportunities to exchange knowledge, reduce feelings of isolation, and gain emotional reassurance. In many community rehabilitation settings, peer support functions as an important source of professional encouragement, particularly when formal supervisory mechanisms are limited or unavailable.

Related to peer support is the use of informal supervision and reflective practice. Several studies indicate that anti-drug social workers engage in regular discussions with senior colleagues or experienced practitioners to obtain guidance on complex cases. Reflective practice enables workers to examine their professional decisions, emotional responses, and intervention strategies. Through reflection, social workers are able to identify strengths and weaknesses in their practice, improve decision-making skills, and develop greater confidence when dealing with difficult clients or organisational challenges.

Professional development emerged as another important coping mechanism. Many anti-drug social workers actively pursue self-learning opportunities to strengthen their knowledge and skills. Given the diverse needs of rehabilitation clients, practitioners often seek additional knowledge in areas such as psychology, mental health, addiction treatment, family intervention, legal procedures, and community resources [62]. Self-directed learning through reading, online training, workshops, and professional networking enables social workers to respond more effectively to complex practice situations and enhances their sense of professional competence.

The accumulation of practical experience also plays a significant role in coping with workplace demands. The reviewed literature suggests that experienced practitioners are often better equipped to manage client resistance, relapse episodes, family conflicts, and administrative pressures [63]. Through repeated exposure to challenging situations, social workers gradually develop practical strategies for communication, conflict management, problem-solving, and case coordination. This experiential learning contributes to greater resilience and professional confidence over time.

At the organisational level, teamwork and collaborative practice represent important coping resources. Anti-drug social workers frequently work within multidisciplinary environments involving community organisations, healthcare providers, law enforcement agencies, and social service departments [64]. Effective collaboration allows practitioners to share responsibilities, access specialised expertise, and coordinate interventions more efficiently. Team-based approaches also provide emotional support and reduce the burden associated with managing complex cases independently.

Interagency networking further assists social workers in addressing resource limitations and service gaps [65]. By establishing professional relationships with external organisations, social workers are better able to access healthcare services, employment opportunities, legal assistance, and welfare support for clients. These collaborative networks not only improve service delivery but also reduce the pressure on individual practitioners to address multiple client needs without adequate support.

Personal coping strategies are also evident throughout the reviewed studies. Many social workers emphasise the importance of maintaining work–life balance as a means of protecting their psychological wellbeing [66]. Engaging in leisure activities, spending time with family, pursuing hobbies, and maintaining social relationships outside work help reduce stress and prevent emotional exhaustion. These activities provide opportunities for psychological recovery and contribute to long-term occupational sustainability.

Emotional regulation is another important personal coping mechanism. Given their frequent exposure to relapse, client resistance, stigma, and emotionally demanding situations, social workers often employ strategies such as emotional distancing, cognitive reframing, mindfulness, and self-reflection to manage stress [67]. These approaches help practitioners maintain professional boundaries while continuing to provide empathetic and effective support to clients. Collectively, these findings suggest that coping among anti-drug social workers is a multidimensional process involving individual resilience, professional development, peer support, and organisational collaboration.

## 4. Discussion

### 4.1. Professional Role Tensions in Anti-Drug Social Work

This review demonstrates that anti-drug social workers occupy a critical yet highly demanding position within drug rehabilitation systems. The findings suggest that their responsibilities extend far beyond routine case management. In practice, they function as information providers, resource linkers, and relationship repairers while simultaneously responding to institutional expectations shaped by surveillance, compliance, and administrative accountability. This places anti-drug social workers in a complex professional role where supportive intervention and regulatory responsibilities frequently intersect. Their practice cannot be understood solely through the tasks they perform but must also be examined in relation to the broader structural and organisational environments in which these tasks are carried out.

The growing policy emphasis on anti-drug social work reflects broader efforts to formalise and strengthen the workforce within community-based rehabilitation systems. Such developments indicate increasing recognition of social workers as key contributors to relapse prevention, social reintegration, and long-term recovery. As community-based rehabilitation continues to expand, the role of anti-drug social workers is likely to become increasingly important in supporting sustainable recovery outcomes and promoting public health objectives.

A central issue emerging from the findings is the tension between professional care and institutional control. Anti-drug social workers are expected to build trust, provide emotional support, facilitate reintegration, and respond to the long-term needs of clients. At the same time, they are often required to work within systems that emphasise monitoring, reporting, and behavioural compliance. This dual expectation can make it difficult to sustain a clearly defined helping role, especially when clients perceive the worker as part of a control-oriented system rather than as an independent professional. In such contexts, trust building becomes more fragile, disclosure may be more limited, and engagement may remain superficial even when the worker is committed to a supportive approach [68,69,70,71].

The findings also suggest that role tension is reinforced by the broad and overlapping nature of anti-drug social work itself. The multiple roles performed by anti-drug social workers create a complex professional environment in which practitioners must continually balance competing expectations. Rather than operating within clearly defined boundaries, social workers frequently move between supportive, coordinative, and regulatory functions. This complexity may contribute to role ambiguity and challenges in maintaining a coherent professional identity, particularly when institutional expectations conflict with client-centred practice principles [23,24]. Similarly, the role of resource linker goes beyond referral and involves active negotiation with service systems to secure access to welfare, healthcare, and rehabilitation support [29,30]. As relationship repairers, social workers are also expected to help rebuild trust within families, partnerships, workplaces, and communities, often in situations where stigma and repeated relapse have already weakened social ties [37,38]. These overlapping expectations mean that anti-drug social workers must constantly shift between functions while trying to preserve a coherent professional identity.

This role complexity becomes especially challenging when clients expect social workers to provide solutions that exceed available institutional resources. Some individuals may hope that workers can secure employment, financial support, housing, or policy flexibility beyond what existing systems permit. When those expectations cannot be met, the disappointment may be directed towards the worker rather than the structural limits of the system. As a result, social workers may find themselves carrying the emotional weight of unmet need while also confronting the practical reality that many barriers to reintegration remain outside their direct control [72,73,74]. This mismatch between need and capacity can reduce perceived effectiveness and contribute to frustration, not because the worker lacks commitment, but because the surrounding system restricts what can realistically be achieved.

Identity ambiguity further deepens these tensions. The findings indicate that anti-drug social workers are often positioned as intermediaries between institutional mandates and client needs. They are expected to advocate, support, and motivate, yet they may also be required to report client behaviour to supervisory bodies or participate in procedures that clients experience as controlling. This contradiction can weaken the therapeutic relationship and create uncertainty about where the worker stands in the eyes of the client [75,76]. Over time, this may undermine professional confidence and blur the distinct contribution of social work within drug rehabilitation. The discussion therefore suggests that role clarity is not merely a matter of job description, but a foundational condition for building trust, preserving professional identity, and sustaining meaningful intervention.

### 4.2. Organisational Constraints and Workforce Pressures

The review findings indicate that the challenges faced by anti-drug social workers are not simply individual burdens but are deeply embedded in broader organisational arrangements. One of the most prominent issues is governance fragmentation. Social workers often operate within systems that involve social work agencies, anti-drug offices, community organisations, and public security departments, each carrying different expectations, reporting lines, and performance indicators. This creates overlapping systems of accountability that may not align with one another, leaving workers to navigate multiple priorities at the same time [59,60,61,62]. In practice, this fragmentation weakens professional autonomy and makes it more difficult for social workers to prioritise client centred intervention.

A related challenge is the growing administrative burden attached to daily practice. The findings show that anti-drug social workers are frequently required to complete extensive documentation, reporting tasks, and temporary assignments that consume a substantial part of their working time [52,53,54]. Although these activities are often justified in terms of accountability and service monitoring, they may reduce the time available for counselling, follow up, family engagement, and other forms of direct support. In anti-drug rehabilitation, where trust building and long term engagement are central, this reduction in relational practice is particularly concerning. Administrative drift does not only make work more tiring. It also changes the substance of the profession by moving effort away from therapeutic engagement and towards bureaucratic maintenance [66,67].

Training limitations further intensify these organisational pressures. The results show that many anti-drug social workers are expected to manage mental health crises, legal vulnerability, chronic illness, and complex family situations, yet they often do so without sufficient access to structured training in psychology, law, and clinical or health related knowledge [55,56]. This gap can reduce confidence and limit the capacity of practitioners to respond effectively to the complex realities of rehabilitation work [63,64]. In some settings, workers appear to rely heavily on informal learning and accumulated experience rather than on systematic professional development. The problem is not a lack of willingness to learn, but an absence of training structures that match the demands of the field.

The dilution of professional identity is also reinforced when workers are required to participate in activities that fall outside the core scope of social work. The reviewed evidence notes that mandatory tasks such as anti-terrorism or anti evil education may draw time and attention away from direct service and professional development [65]. These activities reflect administrative priorities, yet they risk repositioning social workers as general purpose functionaries rather than specialised rehabilitation professionals. When such expectations accumulate, they may weaken the distinctiveness of social work practice and make it harder for practitioners to maintain a clear sense of professional purpose.

Taken together, these organisational constraints suggest that many of the challenges faced by anti-drug social workers are structural rather than individual in nature. While practitioners are often expected to deliver comprehensive and sustained support, their capacity to do so is shaped by broader organisational arrangements, resource availability, and institutional priorities. Consequently, improving service effectiveness requires not only individual competence but also systemic reforms that support professional autonomy, workforce development, and sustainable practice environments. Addressing these structural barriers is essential for strengthening workforce stability, improving service quality, and enhancing long-term rehabilitation outcomes.

### 4.3. Coping Strategies and Workforce Resilience

The findings suggest that anti-drug social workers do not respond passively to workplace challenges but actively employ a range of coping strategies to maintain professional functioning and psychological wellbeing [77]. These strategies operate across multiple levels, including individual, interpersonal, and organisational domains. The presence of such coping mechanisms highlights the adaptive capacity of practitioners working within demanding rehabilitation environments.

Peer support emerged as one of the most important coping resources identified in the reviewed studies. Informal consultation with colleagues provides opportunities for emotional validation, knowledge sharing, and collective problem-solving. This finding is consistent with broader social work literature, which emphasises the protective role of collegial relationships in reducing occupational stress and preventing professional isolation. In environments characterised by high caseloads and emotional labour, peer support may serve as an accessible and sustainable source of resilience.

Reflective practice and informal supervision also appear to play a significant role in strengthening professional competence and emotional wellbeing. Through reflection and consultation, social workers are able to process challenging experiences, evaluate intervention strategies, and improve professional judgement. These practices contribute not only to individual learning but also to the development of a more reflective and adaptive workforce. The findings therefore support calls for more structured supervision systems within community-based rehabilitation services.

Professional learning represents another important dimension of workforce resilience. Many social workers compensate for gaps in formal training through self-directed learning and continuous professional development [78,79,80]. While this demonstrates strong professional commitment, it also highlights systemic shortcomings in workforce preparation. Reliance on individual initiative alone may not be sufficient to address increasingly complex rehabilitation needs. Greater investment in specialised training programmes is therefore necessary to strengthen professional capacity and service quality.

The review further demonstrates the importance of organisational support in facilitating effective coping. Team collaboration and interagency networking enable social workers to share responsibilities, access additional resources, and manage complex cases more effectively [81,82]. These collaborative approaches reduce professional burden and contribute to more integrated service delivery. Organisational environments that encourage teamwork and interdisciplinary cooperation are therefore likely to enhance both workforce wellbeing and client outcomes.

Personal coping strategies, including work–life balance and emotional regulation, also contribute to occupational sustainability [83,84,85,86]. While individual resilience is important, the findings suggest that personal coping should not be viewed as a substitute for organisational support. Excessive reliance on individual coping may unintentionally shift responsibility away from institutions and obscure structural factors that contribute to occupational stress. Sustainable workforce development therefore requires a balanced approach that combines personal resilience with supportive organisational policies, effective supervision, manageable workloads, and ongoing professional development opportunities.

### 4.4. Public Health Implications of Community Based Rehabilitation

The findings of this review have important implications for how anti-drug social work is understood within a broader public health framework. Community based rehabilitation is increasingly positioned as a strategy for relapse prevention, harm reduction, social recovery, and long-term reintegration. The findings of this review demonstrate that anti-drug social workers support rehabilitation outcomes not only through direct intervention but also through their ability to connect clients with essential social, health, welfare, and community resources. As information providers, resource linkers, and relationship repairers, they facilitate access to services and support systems that are critical for sustained recovery. Their contribution therefore extends beyond individual case management and aligns closely with broader public health goals, including social inclusion, harm reduction, relapse prevention, and community wellbeing. By addressing the social determinants that influence recovery, anti-drug social workers play an important role in promoting long-term rehabilitation and reducing social vulnerability among people who use drugs.

At the same time, the findings indicate that the public health value of community-based rehabilitation may be weakened when the workforce responsible for psychosocial support is overburdened, undertrained, and structurally constrained. Fragmented governance, competing institutional demands, and excessive administrative work reduce the capacity of social workers to provide sustained psychosocial intervention. This matters because relapse prevention depends not only on the availability of programmes, but also on the quality and continuity of relationships through which support is delivered. When workers are unable to spend sufficient time with clients, when trust is undermined by role ambiguity, or when burnout reduces workforce stability, the effectiveness of rehabilitation as a public health strategy may be compromised.

The findings also point to the importance of social determinants in shaping rehabilitation outcomes. Clients in anti-drug services often face poverty, stigma, unstable employment, weak social support, and limited access to healthcare. These are not peripheral issues but central factors that influence relapse risk and the capacity for reintegration. The roles identified in the results, particularly those related to resource linkage and relationship repair, show that anti-drug social workers are addressing determinants that sit at the heart of public health concerns [29,30,37,38]. Their contribution therefore extends beyond individual casework and into the wider task of reducing social vulnerability and supporting community wellbeing.

From this perspective, strengthening the anti-drug social work workforce should be seen as a public health investment rather than only an organisational concern. Role clarity, manageable workloads, targeted training, and stable supervision are not simply staff support measures. They are mechanisms through which the quality, continuity, and reach of rehabilitation services can be improved. When social workers are better supported, they are more likely to sustain engagement with clients, coordinate appropriate referrals, rebuild damaged relationships, and respond effectively to the conditions that contribute to relapse. Strengthening the workforce therefore contributes directly to the broader goals of rehabilitation systems, including healthier communities, reduced social harm, and more sustainable recovery pathways.

Although the reviewed studies are situated within the Chinese context, the issues identified in this review resonate more widely with international concerns about governance complexity, burnout, and the need for integrated community responses in substance use services. The discussion therefore supports a broader argument that psychosocial rehabilitation cannot be effective if the workforce responsible for sustaining recovery is treated as secondary to administrative control. A public health approach to rehabilitation requires not only programmes and policies, but also an adequately supported professional workforce capable of translating those programmes into meaningful and continuous human support.

### 4.5. Practice and Policy Recommendations

The recommendations presented below are derived directly from the themes identified in this review, particularly those relating to role ambiguity, governance fragmentation, administrative burden, limited training opportunities, and workforce wellbeing. Addressing these issues is essential for strengthening the effectiveness and sustainability of community-based rehabilitation services.

Targeted and continuous professional development is essential. The findings indicate that anti-drug social workers regularly encounter issues related to mental health, legal vulnerability, chronic illness, family conflict, and social reintegration, yet many lack sufficient formal training in these areas [63,64]. Training should therefore be designed around the realities of the field rather than provided in generic form. Areas such as trauma informed communication, family intervention, legal literacy, health related knowledge, motivational engagement, and case coordination are likely to be especially important. Training should also be linked to practical supervision so that learning can be applied, reflected upon, and adapted to complex case situations.

Organisational reform is needed to reduce administrative overload and protect the time required for meaningful client engagement. Documentation and reporting are necessary, but they should not dominate the work process to the point that psychosocial intervention becomes secondary. More realistic workload allocation, simplification of bureaucratic procedures, and the reduction in non-core assignments would help social workers spend more time on assessment, follow up, family work, and relationship building. These are the very activities that the results identify as central to relapse prevention and long-term recovery.

Supervision should be recognised as a core component of service quality and workforce sustainability. The emotional labour involved in anti-drug social work is substantial, particularly in contexts of repeated relapse, family conflict, client resistance, and limited visible progress [78,79,80]. Without structured opportunities for reflection and support, emotional strain can accumulate and contribute to burnout. Supervision offers more than emotional relief. It provides a space for reflective learning, ethical discussion, case consultation, and the strengthening of professional judgement [87,88,89,90]. In high pressure practice environments, regular supervision should therefore be institutionalised rather than left to informal arrangements.

Stronger interagency collaboration is necessary if anti-drug social workers are to function effectively as resource linkers and community-based rehabilitation professionals. Many client needs cannot be addressed within a single service. Partnerships with healthcare providers, welfare agencies, legal services, community organisations, and employment support systems are essential for improving referral pathways and expanding the real support available to clients. Such collaboration should be backed by clear communication channels, shared responsibilities, and policy support. Without this, social workers may continue to shoulder responsibility for problems that cannot be resolved through individual effort alone.

Taken together, these recommendations suggest that effective anti-drug social work depends on more than personal commitment or individual coping. It requires an enabling environment in which professional roles are clear, training is relevant, supervision is stable, and organisational systems support rather than hinder meaningful practice. If community-based rehabilitation is to achieve its full potential, then the conditions under which anti-drug social workers operate must become a central focus of reform.

## 5. Conclusions

This systematic review highlights the central role of anti-drug social workers within community-based drug rehabilitation as a frontline public health workforce supporting relapse prevention, social reintegration, and long-term recovery. The findings show that their contribution extends beyond routine service delivery. As information providers, resource linkers, and relationship repairers, they play a critical role in helping individuals navigate complex systems, rebuild social connections, and regain stability during recovery. Their work bridges the gap between institutional frameworks and the lived realities of people who use drugs, making them essential to sustaining rehabilitation beyond formal treatment settings.

At the same time, this review demonstrates that the effectiveness of anti-drug social workers is shaped by structural and organisational conditions. Fragmented governance, role ambiguity, administrative burden, limited training, and sustained emotional demands constrain their ability to provide continuous and meaningful support. These challenges contribute to occupational stress, burnout risk, and workforce instability, which may weaken the continuity and quality of rehabilitation services. The findings suggest that without addressing these underlying conditions, the potential of community-based rehabilitation to support long term recovery will remain limited.

From a public health perspective, strengthening community-based drug rehabilitation requires greater attention to workforce sustainability alongside client focused intervention. Clearer role positioning, reduced administrative demands, targeted interdisciplinary training, and structured supervision are essential to support both professional wellbeing and effective service delivery. Although the findings are situated within the Chinese context, the issues identified reflect broader challenges in substance use services internationally. Addressing these challenges is therefore critical to improving relapse prevention, strengthening rehabilitation outcomes, and enhancing the long-term effectiveness of community-based drug rehabilitation systems.

## Data Availability

Raw data will be made available, if necessary, upon request to the corresponding authors.
